# A Landscape of Cancer Initiation and Cancer Stem Cells

**DOI:** 10.3390/cancers17020203

**Published:** 2025-01-09

**Authors:** Masaharu Seno

**Affiliations:** Laboratory of Cancer Stem Cell Engineering, Faculty of Interdisciplinary Science and Engineering of Health Systems, Okayama University, 3-1-1 Tsushima-Naka, Kita-ku, Okayama 700-8530, Japan; mseno@okayama-u.ac.jp; Tel.: +81-86-251-8265

**Keywords:** cancer initiation, point of shift from normal to malignant, radiation, chemicals, genetic aberration, epigenetics, oncogenic viruses, microbiomes, chronic inflammation, cancer stem cells

## Abstract

Exposure to radiation and chemicals, oncogenic viruses, microbiomes, and inflammation are the major events of cancer initiation. DNA damage and chromosomal aberrations are classically considered the main causes of cancer. The recent idea of epigenetics is broadening the concept, including the suggestion that oncogenic virus infection disrupts various intracellular signaling cascades. Chronic inflammation was proposed as the origin of cancer in the 19th century, and the molecular level of events has been made clear with scientific development. Much knowledge of cancer initiation has become available for integration into research. Simultaneously, the presence of cancer stem cells has been identified and characterized. However, the point of shift from normal to malignant still appears obscure even when taking cancer stem cells into consideration. From these points of view, the advent of cancer stem cells and cancer initiation are briefly discussed as the points of shift from normal to malignant in this paper.

## Simple Summary

Cancer initiation is not precisely defined yet. It should be the point of shift from normal/benign to malignant. However, it is still controversial in spite of the abundant accumulation of data on carcinogenesis. Cancer initiation is overviewed here from different viewpoints of genetics, epigenetics, viral/microbial infections, and chronic inflammation. The advent of cancer stem cells is discussed as the point at which a normal cell acquires malignancy.

## 1. Introduction

The epidemiology of cancer was discussed and theorized throughout the last century [1]. Although cancer is mainly considered a genetic disease, the critical factors are composed of complex events. The causes are heterogeneous and complicated, and the effort to link them together still appears challenging.

When cancer is initiated, it is clear that there should be a point of shift from normal to malignant. Continuous exposure to radiation and/or hazardous chemicals, heredity of impaired genes, oncogenic virus infection, chronic inflammation, and chronic diseases are currently conceivable causes. Some of them are directly or closely related to mutations and genetic alterations, including genome translocation as typically found in Bcr-Abl fusion in chronic myelogenous leukemia associated with neutrophilia and thrombocytosis [2]. KRAS2 mutation is another example implying that there may be several critical factors [3]. It initiates pancreatic cancer in pancreatic duct cells. In contrast, it is not directly related to carcinogenesis in normal colon epithelial cells without developing malignancy, while it often results in the progress of cancer in the same cell type in cases where the APC gene has mutated in advance. Some cases are not directly related to mutations or genetic events but apparently epigenetic regulations. This is demonstrated by the acquired unlimited cell growth of human mammary epithelial cells with suppressed expression of p16, of which the promoter was silenced by repeated hypermethylation after 20 passages in serum-free MCDB 170 media [4] and increased the oncogenic activity of WNT5 with a hypomethylated promoter in prostate cancer [5,6]. Familiarly inherited mutations, such as in BRCA1/2, APC, and RB1, are considered to be linked to genetic instability [3]. Unrelated to immediate cancer initiation, these mutations apparently allow the elimination of a tumor-suppressor gene in one allele depending on the microenvironments. It is worth noting that inflammation contributes to mutagenesis and chromosomal instability [7,8]. Even aneuploidy could be associated with inflammation [9].

In this commentary, exposure to radiation and chemicals, oncogenic viruses, and inflammation are separately summarized as the three major events of cancer initiation in Section 1. Each part is supplemented with comments on the relationships with cancer stem cells. In Section 2, the advent of cancer stem cells is summarized, including the results from recent papers on the cancer stem cell models developed from pluripotent stem cells. The generation of cancer stem cells is also discussed, taking the events of cancer initiation into consideration.

## 2. Events of Cancer Initiation

### 2.1. Exposure to Radiation and/or Chemicals

Short-wave ultraviolet light and X-rays are well known to induce covalent linkages via so-called photochemical reactions [10]. This reaction happens between the neighboring bases in the nucleotide sequences, resulting in the formation of a cyclobutene ring of pyrimidine dimers of thymine or cytosine bases in DNA [11]. This dimer causes a distortion in the DNA structure, preventing replication or transcription beyond the site of the dimerization. Although approximately 50 to 100 reactions per second are considered to occur in a skin cell exposed to sunlight, the impaired DNA is usually repaired within seconds by the excision of the nucleotide dimers in humans, followed by the DNA polymerase activity incorporating the nucleotides corresponding to the other strand of the double helix [12]. However, the fidelity of the repairing enzymes is not always perfect. If the repair process fails, mutations can arise within the genome of an organism, having effects on translation such as missense, nonsense, and frameshift, which sometimes lead to the initiation of cancer. For example, when DNA damage occurs in a cell at the G1 phase, the cell cycle is stopped, and DNA replication is postponed until the damage is repaired. Then, a transcription factor, p53, induces the expression of the genes for DNA repair, indirectly inhibiting the cyclin-dependent kinase complex, which is responsible for progressing the cell cycle from the G1 to S phases. If the function of p53 is impaired by some mutations, the cell cycle proceeds without DNA repair, resulting in chromosome rearrangements and gene amplification, which may induce apoptosis or, otherwise, cancer initiation. Thus, the risk of cancer initiation is increased by the accumulation of mutations, which may induce serious lesions of genes, resulting in false transcription, translation, and/or replication [13].

Pyrimidine dimers are considered the primary cause of melanomas in humans. The typical case is xeroderma pigmentosum (XP), which is a rare genetic disease with autosomal recessive inheritance in humans and the common features and symptoms of skin discoloration and the formation of multiple tumors [12,14]. In XP, the genes coding the enzymes responsible for the nucleotide excision of pyrimidine dimers are impaired by mutations, resulting in the high risk of melanoma initiation. Since ultraviolet radiation triggers and enhances the differentiation of melanoblasts into melanocytes that produce melanin in 1 to 2 weeks [15,16], the advent of cancer stem cells derived from melanoblasts may reflect the potential for differentiation into melanoma cells producing abundant melanin.

Exposure to ionizing radiation such as gamma rays, X-rays, and the higher-energy ultraviolet is considered to increase the probability of the initiation of cancer, particularly leukemia [17]. The risks of cancer induction from ionizing radiation are hypothesized to increase linearly with an effective dose of radiation less than approximately 100 mSv [18]. However, it should be noted that there is no model to quantify the level of risk and that natural background radiation, such as sunlight, which is non-ionizing radiation, represents a potentially hazardous case for pyrimidine dimer formation.

Internal doses of iodine-131 have typically been observed to have non-linear effects [19]. High doses of this isotope are sometimes less dangerous than low doses because they kill thyroid tissues before the radiation-induced cancer is initiated. Even when a small number of cells survive and initiate cancer, this can take years to be recognized. Thyroid cancer did not increase in the treatment of Graves’ disease with iodine-131 at high doses, while the absorption of iodine-131 linearly increased the risk of thyroid cancer [20]. Meanwhile, it has been reported that an overdose of ionizing radiation beyond antioxidant capacity may produce reactive oxygen species (ROS), leading to oxidative stress and causing serious damage such as genetic mutations [21,22,23]. On the other hand, ROS act as pleiotropic signal transducers to maintain various biological processes/functions such as mitochondrial electron transfer and NADH oxidase, including immune response involving TLRs, NFκB, JNK, NRF2, p53, and STAT3 [24].

There are many chemicals well known as mutagens and carcinogens, the chemical reactions of which change DNA structure and/or induce a high dose of ROS [24,25,26]. Chemicals naturally or anthropogenically present in the air, water, and soil, such as tobacco smoke [27], beverages containing alcohol [28], arsenic [29], heavy metals [30], asbestos [31], aflatoxin [32], pesticides [33], and aromatic hydrocarbons like benzene and derivatives [34], as well as some anti-cancer drugs [35], are suspected to be carcinogens. Historically, it is notable that Yamagiwa and Ichikawa first demonstrated the induction of tumor formation by applying an extensive amount of coal tar–benzene solution to the ears of rabbits [36].

As described above, once the DNA is damaged, its function is impaired unless it is repaired in a regular manner. Otherwise, the accumulation of damage may lead to multiple changes in genes, resulting in cancer initiation. It is noteworthy that cell death is simultaneously induced, followed by wound healing for tissue repair. However, it is still difficult to recognize the point of cancer initiation, which involves the appearance of cancer stem cells. Cancer does not usually develop immediately since the growth of a cancer stem cell is slow, and it may take months to years before cancer becomes visible in the body.

### 2.2. Oncogenic Viruses

Seven viruses are currently recognized as oncogenic in humans, namely, hepatitis B virus (HBV), hepatitis C virus (HCV), human T-lymphotropic virus (HTLV), human papillomavirus (HPV), Epstein–Barr virus (EBV), Kaposi’s sarcoma-associated herpesvirus (KSHV), and Merkel cell polyomavirus (MCV) [37,38]. The Group 1 classification of the International Agency for Research on Cancer (IARC) includes HIV and not MCV. However, HIV is not discussed here because it mainly acts as an indirect carcinogen in an immunosuppressive manner [39]. On the other hand, MCV is explored here because it is considered to be associated with Merkel cell carcinoma and is included in the class of probable carcinogens, IARC Group 2A [40]. In almost all cases of infection, the viruses disrupt the intracellular signaling pathway and/or have their genomes integrated into the host chromosomes, resulting in cancer initiation after residing in the host for a long period.

#### 2.2.1. HBV and HCV

Chronic hepatitis is induced by the HBV and HCV infection, leading to cirrhosis and hepatocarcinoma [41,42]. HBV is a DNA virus that is divided into four major serotypes (adr, adw, ayr, and ayw) based on antigenic epitopes present on its envelope proteins, and HBx is considered an oncogene product that interacts/associates with various signal transducing molecules such as p53, JUN, JNK, Src, JAK/STAT, RAS, ERK, FOS, CREB, AP-1, ATF-2, and NFκB.

HCV is an RNA virus in which its genome encodes four oncogenic proteins, Core, NS3, NS5A, and NS5B, are encoded [43]. These proteins are reported to activate, modulate, or regulate p53, Raf-1, cyclin-dependent inhibitor p21, c-Myc, AP1, human telomerase reverse transcriptase (hTERT), TGFβ signaling, and so on. HCV infection also induces oxidative stress.

#### 2.2.2. HTLV-1

Human HTLV-1 is a retrovirus that causes adult T-cell leukemia/lymphoma (ATL). Flanked with 5′- and 3′-LTRs, the genome codes the oncogenic protein Tax in the plus and HBZ in the minus strand [44,45]. This protein is considered to play a pivotal role in the initiation of ATL. Tax activates IKK/NFκB signaling, mTOR, CREB, DNA damage response, and so on. In contrast, HBZ antagonizes many of the activities of Tax. HBZ can target the complex of retinoblastoma tumor suppressor protein (pRb) and E2F1 to activate the transcription of the genes primarily responsible for DNA replication and cell cycle progression. HBZ also assists JunD to activate hTERT. Importantly, HBZ was implied to induce T-cell lymphoma and systemic inflammation when expressed in CD4^+^ T-lymphocytes.

#### 2.2.3. HPVs

HPVs are circular, double-stranded DNA viruses consisting of more than 200 subtypes, of which 16, 18, 31, 33, 35, 39, 45, 51, 52, 56, 58, 68, 73, and 82 are considered highly carcinogenic, especially inducing cervical cancer [46]. Among these high-risk subtypes, HPV16 and 18 are well studied, and oncogenes E5, E6, and E7 are described [47,48].

E5 is a transmembrane protein composed of 83 amino acid residues localizing in the endoplasmic reticulum, the Golgi apparatus, the nuclear membrane, and the endosomes. E5 can stabilize epidermal growth factor receptor and stimulate mitogen-activated protein kinase (MAPK) activity, suggesting that it can control cell division pathways, maintaining the life cycle of the virus before the viral genome is integrated into the host. E6 is an 18 kDa protein composed of approximately 150 amino acid residues. E6 also enhances the degradation of p53 and increases the hTERT activity by enhancing the transcription of the telomerase catalytic subunit gene [49]. E7 is a protein of 100 amino acid residues. E7 inhibits pRb, which is responsible for cell cycle regulation and apoptosis. Further, E6 and E7 have been implied to regulate cell proliferation through the mTOR pathway.

Since E6 and E7 are the biomarkers of cervical cancer cells and cancer progression, the viral oncoproteins should play a pivotal role in the oncogenesis of infected cells. However, the detection of HPV mRNA E6/E7 in vivo does not mean that a host has developed cancer. It indicates that the host is infected with HPV, which carries a risk of cancer. In the process of viral genome replication, these proteins can induce all the hallmarks of a cancer cell, namely, uncontrolled cellular proliferation, angiogenesis, invasion, metastasis, and unrestricted telomerase activity evading apoptosis and the activity of growth suppressors. There should be a point of shift from normal to malignant when cancer is initiated.

#### 2.2.4. EBV

EBV is known as human herpes virus 4 and is associated with lymphomas and epithelial cell carcinomas [37,50,51]. EBV first infects naïve B cells, establishing the latency III program to express the viral genes Epstein–Barr nuclear protein antigen (EBNA) 1–6, latent membrane protein (LMP)1, LMP2A, and LMP2B. In this program, the infected cells become proliferative, allowing episomal replication of the virus genome.

Since EBNA-2 and -3 proteins are highly immunogenic, the latency shifts to latency II, which is not immunogenic but expresses EBNA1, LMP1, and LMP2A. LMP1 and LMP2A mimic CD40 and IgG receptors to drive the differentiation of B cells into resting memory B cells. The infected dividing memory B cells switch to the other program, so-called latency I, which only allows the expression of EBNA1. EBNA1 can amplify the expression of NADPH oxidase to induce the production of ROS that damages DNA and chromosomes in the host cells, which develop malignancy as a result. LIMP1 constitutively aggregates and recruits tumor necrosis factor receptor (TNFR)-associated factor and TNFR type 1-associated death domain protein, leading to the activation of NFκB, which is considered to play a pivotal role in initiating lymphoma. NFκB promotes cell survival, upregulating the genes for anti-apoptotic factors A20 and Bcl-2 as well as for a variety of those related to B-cell proliferation and malignancy, including genes related to inflammation such as IL-6, ICAM-1, and LFA-3. LMP2A induces the phosphorylation of AKT, activates the PI3K/AKT pathway, and simultaneously inhibits epithelial cell differentiation in EBV-infected cells, and it is suggested that this process occurs via the same mechanism that contributes to the progression of carcinomas and lymphomas associated with EBV.

Latency I is considered to be associated with Burkitt’s lymphoma and II with Hodgkin’s disease and nasopharyngeal carcinoma. Latency III is associated with AIDS-related non-Hodgkin lymphomas and post-transplant lymphoproliferative disorder due to the most impaired immune system.

#### 2.2.5. KSHV

KSHV is also known as human herpes virus 8 [50]. The KSHV genome encodes viral caspase-8 (FLICE) inhibitory protein (vFLIP), vGPCR, latency-associated nuclear antigen (LANA), replication and transcription activator (RTA), and viral interleukin-6 (vIL-6), which can induce the expression of Notch ligands and receptors, leading to the activation of the pathway. During the progression of Kaposi’s sarcoma, the expression of genes associated with the cell cycle in adjacent uninfected cells appears to be inhibited by the Notch signaling activated by the viral proteins. As a result, the growth of uninfected cells is relatively suppressed, and that of infected cells is enhanced. The infected lymphatic endothelial cells are reprogrammed to mesenchymal cells depending on membrane-type-1 matrix metalloproteinase through the Notch pathway activated by vFLIP and vGPCR. This process is named “endothelial-to-mesenchymal transition” [52].

LANA prevents proteasomal degradation of the intracellular domain of NOTCH1 (ICN), inhibiting the ubiquitination of ICN by F-box/WD repeat-containing protein 7 (FBXW7) in a competitive manner [53]. As a result, ICN can keep stimulating the proliferation of KSHV-infected tumor cells, promoting virus-mediated transformation. Both positive and negative regulation of Notch signaling can provide viral oncogenesis, implying that transformation depends on the framework of the cellular environment and the infected cell type.

As well as Kaposi’s sarcoma, KSHV induces primary effusion lymphoma cells, in which NFκB is constitutively activated by vFLIP directly binding to an inhibitor of NFκB (IκB) kinase (IKK) [54,55].

#### 2.2.6. MCV

MCV is suspected to cause a rare but aggressive skin cancer, which is called Merkel cell carcinoma (MCC) [56]. Approximately 80% of MCC tumors have been found to be infected with MCV. MCV has a double-stranded DNA genome of approximately 5.4 kbp coding characteristic polyomavirus proteins, including a large T (LT) and a small T (sT) antigen and viral capsid VP1 and VP2/3 proteins [57]. LT and sT antigens are alternatively spliced products that have features of oncoproteins similar to the T antigens of other polyomaviruses [58,59]. LT is coded in exons 1 and 2, while sT contains a part derived from the intron in place of exon-2. Therefore, LT has a helicase domain derived from exon-2 that may cause damage to DNA [60], and sT has a region derived from intron-preserving eukaryotic translation initiation factor 4E–binding protein 1 (4E-BP1) hyperphosphorylation, which results in dysregulated cap-dependent translation through the mTOR pathway [56]. Both T antigens have the same amino-terminal domain that is capable of binding to pRb [60] and are probably responsible for transforming healthy cells into cancer cells.

Overall, the common situation among the seven oncogenic viruses is the introduction of foreign viral factors that affect the intercellular signaling and cellular phenotypes. The effects remain for the duration of the infection, often leading to carcinogenesis. This appears like a kind of reprogramming as found in induced pluripotent stem cells [61] when compared to the case of naïve B cells in EBV infection. The viral transformation of a cell could be traced to the reprogramming of a cell, generating a cancer stem cell as the point of cancer initiation.

### 2.3. Microbiomes

The links between microbiomes other than oncogenic viruses and cancer are repeatedly reviewed [62,63,64,65]. Due to infection, immunogenic responses are evoked against the toxins and/or metabolites produced by the microorganisms, as well as the bacterial antigens, including endotoxins, such as lipopolysaccharides and flagellins, resulting in inflammation. For example, T-helper 17 cells activated by pathogenic toxin are suggested to mediate colitis in the gastrointestinal tract, resulting in the activation of colon-specific STAT3 that may induce multiple intestinal neoplasia related to the APC mutant. Butyrate produced by microbials can stimulate naïve T cells and dendritic cells to differentiate into regulatory T (Treg) cells. Butyrate inhibits histone deacetylases and epigenetically activates the forkhead box P3 master regulator, which is a specific marker of natural Treg cells. Attenuation of chronic inflammation by infiltrating Treg cells may allow the initiation of cancer.

When suppressing apoptosis under chronic infection conditions in a weakened immune system, the pathogen accumulates intracellularly, inactivating the retinoblastoma protein and modulating the expression of proteins in the Bcl-2 family. This allows cells to transform and become carcinogenic, evading the process of cell death. Chronic bacterial infection responsible for periodontitis in the oral cavity sometimes allows infiltration of immune cells, which produce reactive nitrogen and oxygen species, resulting in the induction of DNA damage. Moreover, increased levels of the bioactive molecules may promote tumor progression.

Metabolism via bacteria producing potential carcinogens is another possible mechanism. For example, microbes present in the oral cavity convert ethanol into acetaldehyde, which is mutagenic and carcinogenic.

### 2.4. Inflammation

Inflammation can be defined as a protective host response by immune cells and blood vessels displaying a complex biological response against harmful stimuli such as exposure to irritants/radiation and pathogen infection. This response involves damage to tissues and cells, usually followed by wound healing [66,67]. Namely, this response of inflammation includes the processes to eliminate the initial cause of cell injury, to clear out dead cells, and to repair damaged tissues. During the course of processes, immune cells are activated against the stimuli, secreting inflammatory factors such as cytokines and chemokines, followed by growth factors from the surrounding cells to stimulate the growth, differentiation, and motility of the residual cells to repair the damaged tissues in an autocrine and/or paracrine manner.

Thus, inflammation can be a defensive mechanism to protect tissues against injury. It is “acute” if it occurs immediately upon injury, lasting only a few days or up to 2–6 weeks [68]. When inflammation lasts for months or years due to the insufficiency of the defensive mechanism, including autoimmune disorder, it is “chronic” [69]. Macrophages, lymphocytes, and plasma cells dominate in chronic inflammation and neutrophils in acute. Diabetes, cardiovascular disease, and allergies are examples of diseases mediated by chronic inflammation. It is noteworthy that obesity involves the mediators that are promoted in chronic inflammation [70].

Cancer was first hypothesized to originate at the sites of chronic inflammation by Rudolf Virchow in 1863 [71]. Nowadays, we know that chronic inflammation involves various ligands and receptors of cytokines, chemokines, and growth factors, including steroid hormones, which are responsible for the transduction of intercellular signals like MAPK, PI3K, GPCR, and NFκB, as described above, affecting the events of cell survival, growth, and differentiation [72]. Considering that the signals are constitutively activated in a chronic situation for a long period, it is conceivable that the cells adapt to the shifted setpoint of homeostasis, changing the phenotype, namely, initiating cancer. Simultaneously, it is also conceivable that the undifferentiated progenitor cells localized in the damaged tissues are stimulated to repeat self-renewal and differentiation like stem cells and to acquire malignancy characterized as cancer stem cells. We have recently demonstrated the induction of cancer stem cells from normal pluripotent stem cells exposed to fibroblast growth factor 2 (FGF2) and prostaglandin E2 for 4 weeks in vitro [73,74]. This capacity of mediators in inflammation implies the initiation of cancer under chronic inflammation conditions. In the first experiments published in 1916, chronic inflammation should have been induced in the rabbit ears due to the extensive application of irritating solution for a long period [36].

The most likely points of cancer initiation are summarized in this section. It should be noted that exposure to radiation and/or chemicals may induce injury in tissues, such as burns followed by inflammation. Infection with oncogenic viruses also induces inflammation if the virus expresses immunogenic antigens. If the mutations are in the genes coding extracellular surface molecules, the amino acid changes could be the targets of the immunogenic response, leading to inflammation. Mutations occurring in cytoplasmic molecules, such as tyrosine kinase domains of receptors, KRAS2, PI3KCA, and BRAF [3], could be related to the inflammatory signaling cascades leading to cancer initiation if they occur before the advent of cancer stem cells. These causes of cancer initiation are feasible means of affecting genomic function with/without mutations and chromosomal rearrangements, though it still appears challenging to specify and identify the actual causes of cancer initiation and separate each factor from the complex effects.

## 3. The Advent of Cancer Stem Cells as the Point of Cancer Initiation

The concept of cancer stem cells has repeatedly been reviewed [75,76,77,78,79]. Cancer stem cells are currently defined by the potential for self-renewal, differentiation, and tumorigenicity. However, the process of the advent of a cancer stem cell is still controversial.

It is worth noting that KRAS2 mutation initiates cancer in the pancreas from the point of mutation. Pancreatic acinar cells actively partake in metaplasia of a ductal cell phenotype in acute and chronic inflammation, indicating a significant link with pancreatic ductal adenocarcinoma [80]. Acinar–ductal metaplasia can represent transdifferentiation through an intermediate phase, which potentially consists of progenitor cells. It is conceivable that KRAS2 mutation occurs in the progenitor cells, potentially driving the transformation of cells into cancer stem cells and being responsible for the development of neoplasia from metaplasia.

Since cellular reprogramming is possible via transient expression of some genes for transcription factors, such as OCT4, SOX2, KLF4, and c-MYC [61], constitutive genetic aberrations like mutations or chromosomal translocations are not always necessary, and transient epigenetic regulation or overstimulation of intracellular signaling cascades is sufficient. In this context, oncogenic virus infection and epithelial-to-mesenchymal transition are conceivable explanations for the advent of cancer stem cells even from normally differentiated mature cells.

Simultaneously, it is necessary to consider the idea of direct reprogramming in viral infection [81]. FGF2 and an artificial retrovirus, γ-retroviral plasmid, promoted the conversion of non-neuronal cells to Dcx^+^ neurons in the adult mouse neocortex [82]. Further, viral vectors conveying reprogramming factors could induce off-target effects such as the ubiquitylation of histone H2A and H2B, which has been linked to both the activation and silencing of gene transcription, resulting in the induction of cancer [83]. Several viral infections, including the HPVs, HBV, EBV, and HCV, promote the aggressiveness of cancer by encouraging the development of CSC features [84].

However, it has not been demonstrated that normal mature cells, namely, fully differentiated cells, are directly reprogrammed to cancer stem cells through the transient process. On the other hand, embryonic stem cells and pluripotent stem cells have been shown to convert into cancer stem cells in the microenvironment of cancer-derived cells or factors related to inflammation [72,73,74,85,86]. Especially, the importance of epigenetics was suggested as CpG islands were hypomethylated to enhance the expression of the genes in cancer stem cells developed using the same approach [85]. Furthermore, epimutations, such as DNA methylation, histone modifications, and non-coding RNAs, have recently been described as being transmitted across generations [87]. Very recently, perturbed epigenetic dysregulation was also shown to induce cancer in the absence of driver mutations via a reversible depletion of Polycomb proteins in *Drosophila* [88]. It would be conceivable to assume that genetic alterations, such as mutations, translocations, and deletions, which are responsible for the cellular transformation, take over the inherited epigenetic dysregulations during the long period as adaptations.

**Figure 1:**
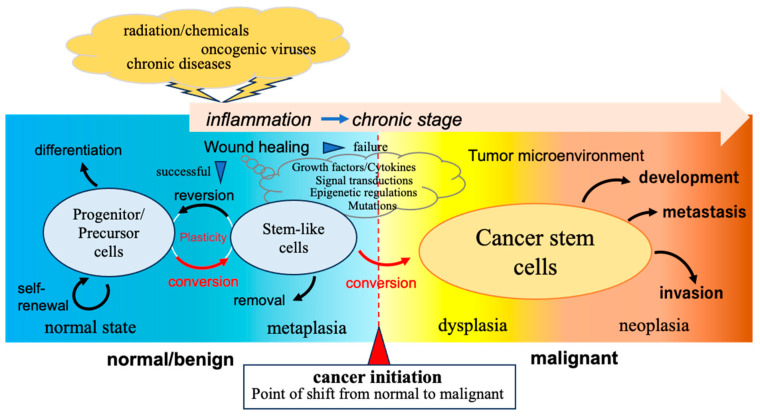
A schematic drawing of cancer initiation as the point of shift from normal/benign to malignant. Progenitor/precursor cells are converted into cancer stem cells under the influence of chronic inflammation due to the failure of wound healing.

It appears necessary to take the concept of cellular plasticity into consideration [89,90]. Every normal tissue has progenitor or precursor cells enabling regular replacement via differentiation into mature cells, which are stable without proliferation but mortal, undergoing senescence to death. Normal enteroendocrine/tuft cell precursors in intestinal crypts have been shown to act as facultative stem cells supporting regeneration after injury [91]. This alteration was characterized by fetal-like conversion and possible metaplasia-like transformation through single-cell transcriptomic analysis. The plasticity between precursors and stem cells in the intestinal epithelium is a conceivable research target in normal and neoplastic contexts in which the shift point from normal to malignant should exist. Hence, these undifferentiated cells could be a target for conversion into cancer stem cells under the conditions described above. It is important to know how much plasticity is necessary for a cell to become a cancer stem cell responding to the stimuli. An overall context of this commentary is summarized in Figure 1.

However, further study on the critical point of stemness loss in cell plasticity is required. If the cells with stemness have sufficient plasticity to respond to the cancer-inducing niche, it is conceivable to consider the advent of a cancer stem cell as the cancer initiation. Following this idea, it is more feasible to explain the heterogeneity of tumor tissues as a hierarchy of cancer-associated cells, such as endothelial cells, cancer-associated fibroblasts, and tumor-associated macrophages, with cancer stem cells as the apex [72,85,92,93]. This way of thinking leads to the concept of neoplasia developing from a cancer stem cell to a tumor tissue that could be compared to a tissue or body developing from a somatic stem cell or an embryonic stem cell.

## 4. Conclusions

While genetic mutations are not always necessary in cancer initiation, both exposure to radiation/chemicals and oncogenic virus infection can lead to inflammation, which appears to be the main cause of carcinogenesis. Immunocompromised conditions should enhance the chronic stimulation of undifferentiated cells in wound healing, resulting in cancer initiation. Thus, cancer initiation can be described as a point of shift from normal to malignant in the advent of cancer stem cells, which might be the origin of the heterogeneity of developing tumor tissues.
